# Letter from S. S. Satchwell, M. D.

**Published:** 1861-08

**Authors:** S. S. Satchwell

**Affiliations:** Paris


### Letter from S. S. Satchwell, M. D.

Paris, February 20th, 1861.

Charles E. Johnson, M. D:

*Dear Doctor:*—France, with a population of nearly forty millions, and a medical profession of unrivalled professional devotion, has only four medical schools, while our own country contains some fifty, if I mistake not. The great Metropolitan school is, of course, situated here, and the remaining auxiliary schools are at Strasburg, Montpelier, and Lyons. Within the last year or two no one can enroll his name at these Medical schools as a pupil, without presenting two Diplomas—one of Bachelor of Arts, and the other as Bachelor of Science. But we take into our offices as medical students, and our Colleges graduate them as Doctors, men who have never been through the elementary spelling book, nor ciphered as far in Daboll's arithmetic as the good old Rule of Three. The whole number of medical students attending lectures each year at these French schools average about two thousand. How many there are every year in attendance upon our hosts of Medical Colleges, and how many graduates they annually turn out upon the body politic, I shall leave for you to figure up for the benefit of our readers. Here the supply is only equal to the demand, and the profession is not degraded by the ignorance and demagogueism of ambitious medical demagogues and pettifoggers; but Doctors are licensed by government upon the recommendation of an able and impartial profession.

In America, the supply far exceeds the demand, and the Colleges have prostituted the profession, and caused an American Medical Diploma in Europe to be regarded more as an evidence of reproach and ridicule than as a mark of merit and capacity in those holding it. It causes me to feel chagrined when I thus see an American Diploma regarded as so much waste paper, and you need not then wonder that I should continue an advocate of American medical reform. No man can practice medicine or surgery in France without he has a French Medical Diploma, and no man can get that Diploma without studying seven years and sustaining an approved and searching examination before an impartial tribunal. So that the croakers at our North Carolina Medical Board of Examiners, an institution entitled to the support of every good man, would find but little sympathy here in their opposition to this professional necessity. They would find the doors, to practice here, closed against them, not more by professional sentiment than by a due appreciation in the community of health and life. Though the time required here for study and Hospital service extends to seven years, many are nevertheless unable even then to graduate, so trying is the ordeal of the examination, and have to be rejected or put back for a longer study. But in our country a man can graduate in medicine at short notice and without any other qualification than the payment of his lectures, even if his time of pupilage is passed outside of College walls, in extravagance and dissipation. Here he is examined thoroughly, at stated periods during his whole course, and no royal blood nor family influence can secure his Diploma, but he must rely alone upon scientific attainments and intellectual strength. And the same holds true in relation to the filling of any medical vacancy of honor or profit in the various Hospitals, Medical schools, or other public institutions.

It is done by a system of *concouvring* which gives to the poor and obscure the same chance and rights as the wealthy and influential. Our boasted republican system, as practiced in our pubic medical institutions, generally departs from this true and just policy and dispense their honors and profits from less worthy motives. With us kissing goes by favors and by considerations connected with popular elections. Such dispensations are made from calculations of wealth and political qualifications, and in a vast majority of instances, merit and intellect are ignored.

I am running this brief comparison more in sorrow than with pleasure, knowing that in most cases truth does more good by being told than suppressed, and hoping that every drop poured into the rising tide of medical reform at home will not be without its weight in aiding to suppress the prostituting influences of our system of medical education, so prolific of medical nuisances. A physician here has no need of even putting his name over his office door or on his door of residence. He merely announces that a graduated Doctor can be had within, and this is as good a passport to public patronage, under the operation of the system here recognized, as if surrounding himself with all the captivating paraphernalia of our most showy practitioners, whose means to secure practice, would, in fact, if attempted in Paris, consign them to shame and disgrace. The attempts of American physicians sometimes made here to catch the popular breeze by showy means more popular elsewhere, only excites the laughter and ridicule of the community and the neglect of the profession. However fond of gaiety, and of glory, and of war, the French people are, it is equally true that they will not confide their health and lives to the treatment of incompetent medicel men. The French government, directed by that extraordinary man, Louis Nopoleon, places a proper estimate upon human life and comfort in all its relations to the medical profession; and the Emperor is constantly developing plans for the encouragement of medical science, and the comfort of the sick, with an energy of purpose and a minuteness of detail that is most useful and gratifying. I should be glad to see our own government and laws enter more heartily into this great work, and our own people rising to a higher appreciation of its importance and necessity.

The remarks so often made by the ignorant and designing, that quacks and quackery flourish most where medical science has most advanced, is incorrect. Paris is in proof of what I say. Quacks there are here, of course, because they are a part of that great humanity which spreads itself over the whole world. But I am convinced that they are comparatively more few in number and make less in proportion than in any other city. The celebrated quack negro doctor, who flourished here a number of years ago with his panacea for cancer, and who was allowed to practice by special enactment, was at length arrested for malpractice, and is now expiating his crimes in hard prison labour as the reward of his rascality and destruction of human life.

It has been said in support of our existing system of manufacturing Doctors tnat it is unavoidable, as the large majority of our medical students are poor and unable to make a greater outlay of time and money in preparations for practice. If the remark be true then, such men should never enter the profession, because all admit that human health and life should not be confided to incompetent hands. But it is not true. My own observations teaches me that the great mass of our medical students are superior in pecuniary resources to the French students who have to support a long apprenticeship of seven years before commencing practice, even after a Diploma of art and science, previous to commencing medicine. The dress and habits, and manner of living of the throngs of students who daily occupy the seats of the lecture rooms here, are evidence of this. I know of scores of medical students here who are toiling on in poverty from year to year, compelled to subsist, a great many of them, on from fifteen to twenty-five cents a day. It is more the luxury and idleness, and extravagance of our young men, than their indigence and necessities, that are filling the profession to overflowing with inefficient members.

I have before referred to Chassaignac, and to the Hospital Larraboissier as a favorite among the valuable Cliniques, medical and surgical, of this wonderful city. You will remember Chassaignac as the inventor of the Ecrasseur, which was introduced into our country a number of years ago. If, like many of the French physicians and surgeons, he has his crochet, and like so many others carries his favorite idea to an extreme, and thus sometimes becomes too enthusiastic in its praise, and too promiscuous in the application of what in its place is really a good and practical instrument, we can pardon him; for to see him, as I have today, remove the entire tongue, hemorrhoidal and erectile tumors, and amputate the neck of the uterus, not only speedily, but without the loss of a drop of blood, covers a multitude of heterodoxies. Its construction and operation is simple. A kind of metallic chain, with short but solid links is thrown around the base of the tumor, or by the aid of a trocar and canula carried through any of the soft tissues. By a simple mechanism, the arc described by the chain is gradually lessened, and the tissues enclosed by it are separated by the force of the pressure. No ligature is needed in operating, as the passage of the chain through vessels causes such a retraction of the coats as renders the ligatures unnecessary. I believe that in operations upon parts where the eye cannot well reach, as in those about the tongue, rectum, etc., this Ecrasseur is an instrument of great value.

Chassaignac's "drainage" is a revival of the old doctrine of the seton, as his opponents, though denied by himself, contend. With a long or short trocar, according to the parts diseased, he plunges it, lined with a canula, through the abscess and out again through the integument. With the simplest arrangement, no injury can be done to the vessels and nerves involved. Withdrawing the trocar, he passes through the canular left behind a tube of india rubber, perforated with holes, for the escape of matter, and ties the two ends together. Thus the serum or pus gradually escapes, the parts collapse as the matter is poured out, and the introduction of air is prevented. He bases the value of his "drainage" upon its ability to exclude air, holding, as he does, the theory that the admission of air into abscesses causes a decomposition of pus, which, acting as a poison, is absorbed into the system, giving rise to depression of the vital forces, irritation, and hectic fever, etc. Nelatin, the best surgeon here, uses this "drainage," and this is a high endorsement. On the other hand, there are those among them, that excellent surgeon and learned critic, Malgaigne, who deny that the contact of air with pus causes a new formation that can injure the system, and therefore, such men reject the practice of Chassaignac. When we consider the danger of opening large abscesses in depraved constitutions, or in delicate and scrofulous subjects, and their tendency to spread and to burrow into the bones and other hidden places of the organization, and thus, in their many liabilities to destroy life, we cannot fail to hail with approbation any new method likely to be the most successful in curing them and in saving life.

Yours, etc.,

S. S. SATCHWELL.

